# Rare germline variants in DNA repair genes and the angiogenesis pathway predispose prostate cancer patients to develop metastatic disease

**DOI:** 10.1038/s41416-018-0141-7

**Published:** 2018-06-19

**Authors:** Martina Mijuskovic, Edward J. Saunders, Daniel A. Leongamornlert, Sarah Wakerell, Ian Whitmore, Tokhir Dadaev, Clara Cieza-Borrella, Koveela Govindasami, Mark N. Brook, Christopher A. Haiman, David V. Conti, Rosalind A. Eeles, Zsofia Kote-Jarai

**Affiliations:** 1 Oncogenetics, Division of Genetics and Epidemiology, The Institute of Cancer Research, London, SW7 3RP UK; 2 Department of Preventive Medicine, Keck School of Medicine, University of Southern California/Norris Comprehensive Cancer Center, Los Angeles, CA 90015 USA; 3 The Royal Marsden NHS Foundation Trust, London, SW3 6JJ UK

**Keywords:** Cancer genetics, Cancer genetics

## Abstract

**Background:**

Prostate cancer (PrCa) demonstrates a heterogeneous clinical presentation ranging from largely indolent to lethal. We sought to identify a signature of rare inherited variants that distinguishes between these two extreme phenotypes.

**Methods:**

We sequenced germline whole exomes from 139 aggressive (metastatic, age of diagnosis < 60) and 141 non-aggressive (low clinical grade, age of diagnosis ≥60) PrCa cases. We conducted rare variant association analyses at gene and gene set levels using SKAT and Bayesian risk index techniques. GO term enrichment analysis was performed for genes with the highest differential burden of rare disruptive variants.

**Results:**

Protein truncating variants (PTVs) in specific DNA repair genes were significantly overrepresented among patients with the aggressive phenotype, with *BRCA2*, *ATM* and *NBN* the most frequently mutated genes. Differential burden of rare variants was identified between metastatic and non-aggressive cases for several genes implicated in angiogenesis, conferring both deleterious and protective effects.

**Conclusions:**

Inherited PTVs in several DNA repair genes distinguish aggressive from non-aggressive PrCa cases. Furthermore, inherited variants in genes with roles in angiogenesis may be potential predictors for risk of metastases. If validated in a larger dataset, these findings have potential for future clinical application.

## Introduction

Prostate cancer (PrCa) is the most common malignancy diagnosed in men living in the developed world and responsible for over 250,000 deaths per year worldwide.^1^ Family history is a strong risk factor for the disease, with twin studies confirming a large contribution by genetic factors.^2,3^ The majority of PrCa cases are diagnosed with intermediate risk disease, although an appreciable number of individuals develop metastatic disease with low survival rates.^4,5^ In order to simultaneously limit overtreatment whilst ensuring early diagnosis of potentially aggressive and lethal cases, it is critical to identify genetic factors predictive of clinical outcome.

Few heritable factors predictive of aggressive PrCa have been identified to date. Although common variants identified thus far explain over a quarter of the familial relative risk of PrCa,^6^ GWAS subset analysis of aggressive disease has failed to find loci specifically associated only with the aggressive phenotype.^7^ We have previously presented evidence that *BRCA2* is a moderate penetrance gene contributing to young-onset disease with a significantly more aggressive clinical course.^8–10^ Furthermore, loss of function mutations in a small number of additional DNA repair genes have been demonstrated to predispose to familial PrCa and are associated with more aggressive phenotypes including metastatic disease.^11–13^

We hypothesised that additional rare germline variants exist that are predictive of poorer prognosis and could improve clinical management of the disease. However, due to the large number of neutral rare germline variants carried by each individual, detection of causative variants is challenging. In an attempt to enrich for rare variants that predispose to the aggressive disease outcome, we designed a case–case study that sampled the extremes of the PrCa phenotype. Our cohort compared young onset, metastatic patients against cases with older onset, indolent disease. We performed whole exome sequencing to identify genes and biological processes with the highest differential burden of disruptive rare variants, which may in turn represent a signature of aggressiveness.

## Materials and Methods

### Study design and sequencing

Germline DNA samples for 144 aggressive (metastatic, diagnosed age<60) and 144 non-aggressive (Gleason score <7, tumour stage T1-2b, no nodal spread or metastases, diagnosed age ≥60) PrCa cases from the UK Genetic Prostate Cancer Study (UKGPCS)^14^ were obtained from whole blood and distributed on three 96-well plates for DNA library preparation and sequencing. To minimise any potential for case–case confounding caused by batch effects, samples were block randomised based on case status and DNA extraction method. DNA samples were fragmented using a Covaris E220 Ultrasonicator and exome sequences enriched using Agilent SureSelectXT2 Human All Exon V5 baits, in 36 pools (8 samples/pool) using 7 bp molecular barcodes. Pools were sequenced on an Illumina HiSeq 2500 instrument (v4 chemistry, 2 × 100 bp reads).

### Variant calling and annotation

Paired end reads were adaptor-masked using Cutadapt 1.5^15^ and aligned to the GRCh37/hg19 reference genome using BWA-MEM 0.7.10.^16^ Variants were jointly called across all samples using GATK 3.5,^17^ following the specified best practices (https://software.broadinstitute.org/gatk/best-practices/). Analysis was restricted to the exome capture regions plus additional 100 bp padding. Variant annotation was performed using wAnnovar,^18^ Oncotator 1.8^19^ and WGSA^20^ (Amazon EC2 cloud, AWS community instance: WGSA055-ubuntu-800G). Combined Annotation Dependent Depletion (CADD) scores^21^ were used to predict deleteriousness of single nucleotide variants and indels. Transcript annotation was taken from the Oncotator pipeline using the transcript list giving priority to known clinical protein changes (Feb 2016). GENCODE (Version 19 - July 2013 freeze, GRCh37 - Ensembl 74) was used as the reference transcript set.

### Variant filtering and sample quality control

Prior to genotype calling, samples were assessed for sufficient coverage (>80% of bases at ≥20 × sequencing depth) and low contamination ( <10%), as estimated by VerifyBamID 1.1.^22^ Samples not achieving these quality thresholds were excluded from further analysis (Supplementary Table 1). After genotype calling, variant quality control was performed in two stages; pre-sample (to exclude low quality variant calls prior to sample QC) and post-sample (to remove additional monomorphic or low coverage variants in the final post-QC sample panel). During pre-sample QC, variants were first filtered to restrict to coding and splice site contexts according to the Oncotator annotation. Subsequent filtering was performed according to genotype quality (95% at Quality Score ≥Q20), read depth (95% at ≥8), GATK VQSR (99.9 tranche) and missing data (<5%). Based on the filtered variant set, samples were assessed for outlying genotype call missingness, excess heterozygosity and discordant sex information using PLINK 1.9.^23^ Divergent ancestry was identified through Principal Component Analysis in conjunction with 1000 Genomes data^24^ (Supplementary Figure 1) and duplication/relatedness were assessed using the SNPRelate Maximum Likelihood Estimation method.^25^ Samples with heterozygosity >3 × standard deviation of the mean and/or mixed ancestry were excluded. After exclusion of samples that failed quality control, final post-sample variant filtering was performed to exclude monomorphic variants and those with >5% missing data. Outliers from Hardy–Weinberg equilibrium in the non-aggressive set were detected with GWASTools^26^ and removed at *P* < 10^−5^ (Fisher’s exact test). After exclusion of data from 8 cases due to mixed ancestry, high heterozygosity or insufficient sequencing coverage, the final sample set consisted of 139 aggressive and 141 non-aggressive cases, and 150,787 coding or splice site variants that passed QC (Table 1, Supplementary Table 1, Supplementary Table 2).

**Table 1:** Clinical characteristics of PrCa cases that passed quality control

	Non-aggressive	Aggressive
Age range		
≤54	0	51
55–59	0	88
60–64	56	0
65–69	50	0
≥70	35	0
M Stage		
M0	141	0
M1	0	139
N Stage		
N0	141	27
N1	0	63
Nx	0	49
T Stage		
T1	84	8
T2	57	11
T3	0	67
T4	0	42
Tx	0	11
Gleason score		
≤6	141	8
7	0	26
≥8	0	79
Unknown	0	26

M Stage indicates the presence or absence of distant metastases and N Stage indicates nodal spread; 1 = present, 0 = absent, x = unknown. T Stage refers to the tumour staging according to the TNM staging system

### Testing for batch effects and case-case confounding

Confounding was assessed through principal component (PC) analysis, using a general linear model to test the association of PCs with the case and batch status. The first 10 PCs were tested, with no association found for either case or batch status. Differences in genotype call rates between cases were assessed using PLINK 1.9.^23^ One-way ANOVA was performed to test for association of ‘missingness’ rate with sample preparation batch. Neither batch effects nor case-case confounding were detected (Supplementary Figure 2).

### Variant and gene-based association tests

Association of individual variants with case status was tested using logistic regression with Firth correction using the rv package^27^ in R and a Bonferroni adjusted significance threshold (*P* < 3.3 × 10^−7^, for 150,757 tested variants).

To assess the potential association between gene-level burden of rare disruptive variants with phenotype, potentially damaging variants were grouped into two categories. Tier 1 (predicted deleterious) were protein truncating variants (PTVs; frameshift indels, stop gain and splice variants) and Tier 2 (predicted damaging) all non-truncating variants with CADD score ≥20 (Supplementary Table 2).^21^ Rare variants were defined as those with MAF <1% in the healthy non-Finnish European population, as reported by the Exome Aggregation Consortium (ExAC).^28^

Gene-level association analyses were performed separately on Tier 1 only, or Tier1 and Tier 2 variants combined, using Fisher’s exact test (PLINK 1.9) or SKAT-O.^29^ The exome-wide significance threshold was set at *P* < 1.4 × 10^−6^, based on 17,658 tested genes and 2 variant sets.

### Gene set association analyses

Gene set analyses were performed separately on Tier 1 only, or Tier1 and Tier 2 variants combined, using Bayesian Risk Index^30,31^ and SKAT-O^29^ methods. BROCA gene set analysis was conducted based on the expanded BROCA cancer risk gene panel of 60 genes.^32^ DNA repair gene set analyses were performed using 177 curated DNA repair genes and pathway groupings.^33^ Hallmark gene set analyses were conducted according to the 50 gene sets curated to represent specific biological processes in the Molecular Signature Database (MSigDB) Hallmark Gene Set Collection (http://software.broadinstitute.org/gsea/msigdb/collections.jsp).^34^

### GO term enrichment analysis

For GO term enrichment analysis, we selected genes with the highest differential burden of disruptive (Tier 1 and Tier 2) variants between aggressive and non-aggressive cases. Genes were ranked based on collapsing burden count odds ratios (ORs) of >2 or <0.5 and filtered with an additional criterion of a minimum count difference of 3 disruptive variants between groups. The enrichment analysis of biological processes and molecular functions was performed using AmiGO,^35^ PANTHER Overrepresentation Test (2016-07-15 release) and GO Ontology database annotation (2016-10-27 release), applying a Bonferroni adjustment to correct for multiple testing.

## Results

### Study design and whole exome sequencing

To investigate the genetic signature of aggressive PrCa risk through a case–case analysis, we performed extreme phenotype sampling^36^ of patients from the UK Genetic Prostate Cancer Study (UKGPCS), in which ~90% of patients had clinically presenting disease at diagnosis.^14^ We selected 144 aggressive and 144 non-aggressive cases for whole exome sequencing. All cases were of self-reported European ancestry and unrelated. The criteria for defining aggressive PrCa cases were metastatic disease combined with early age of onset ( <60 years), while the non-aggressive cases were later onset (≥60 years) and had low risk clinical presentation (Gleason score <7, tumour stage T1-2b, no nodal spread or metastases).

After exclusion of data from 8 cases due to mixed ancestry, high heterozygosity or insufficient sequencing coverage, the final sample set consisted of 139 aggressive and 141 non-aggressive cases (Table 1, Supplementary Table 1). In total, 150,787 quality filtered coding or splice site variants were called across these samples, of which 97,800 were rare (minor allele frequency, MAF,<1%). 4240 of the rare variants were predicted to be protein truncating (Supplementary Table 2). The median number of rare PTVs per individual was 29 (range 16–44), with no significant difference in overall PTV burden between aggressive and non-aggressive phenotypes. The numbers of rare germline PTVs within our sample cohort are in agreement with observations for the general UK population.^37^

### Variant and gene level association analyses

We first assessed association of all coding variants individually and collapsed at the gene level with the extreme phenotypes. Single variant tests were conducted for all variants with no MAF filter applied, whilst gene level burden tests were performed using rare (MAF<1%) variants for Tier 1 (PTVs) or Tier 1 plus Tier 2 (non-truncating variants with a CADD score ≥20) variants separately. As expected due to the modest sample size, no individual variant or gene was associated with aggressive status at a statistically significant level after adjustment for multiple testing (data not shown).

### Bayesian analysis of rare variants in DNA repair genes

**Fig. 1:**
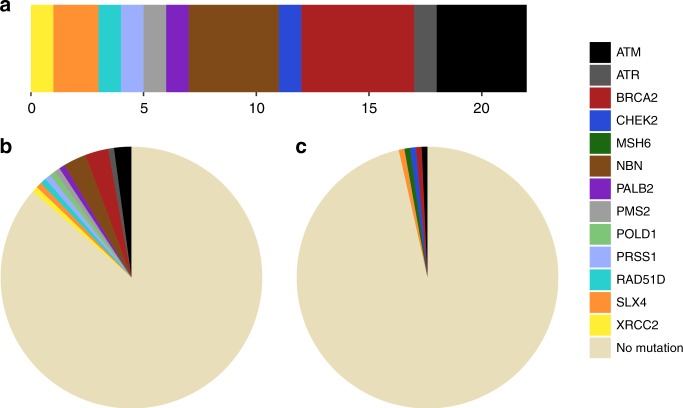
Proportion of rare PTVs identified in the BROCA gene set. **a** Relative frequencies of Tier 1 mutations identified in the combined sample cohort by gene. In total, 22 rare protein truncating variants in BROCA panel genes were identified. **b** Proportion and relative frequencies of germline BROCA panel mutation carriers among metastatic cases. 18 BROCA PTVs, representing 11 unique genes, were identified in 17 patients from the aggressive cohort (12.2%). One individual was a carrier for 2 BROCA PTVs, both in *NBN*. **c** Proportion and relative frequencies of germline BROCA panel mutation carriers among non-aggressive cases. 4 BROCA PTVs, each in a unique gene, were identified in non-aggressive cases (2.8%)

We subsequently conducted analyses at the gene set level, using previously curated gene collections linked to either specific biological pathways or disease in the literature. We have previously shown that deleterious mutations in the original 22 gene BROCA panel of high and moderate risk genes involved in cancer predisposition, primarily focussed on hormone-driven breast and ovarian cancers,^32^ are associated with aggressive PrCa in a familial cohort.^11^ In this study, we identified 22 rare Tier 1 mutations in the extended 60 gene BROCA panel (Table 2 and Fig. 1). These mutations were present in 17 metastatic patients (12.2%) and only 4 non-aggressive cases (2.8%). *BRCA2* (5 cases; 2.9% of metastatic and 0.7% of non-aggressive cases), *ATM* (4 cases; 2.2% in metastatic patients and 0.7% in non-aggressive) and *NBN* (4 mutations identified in 3 cases; 2.2% of metastatic cases and no carriers among the non-aggressive cases) were the most frequently mutated genes, with one metastatic patient a carrier of two Tier 1 BROCA mutations, both in *NBN*. 17 of the Tier 1 BROCA variants in our cohort have been previously reported in ExAC and/or ClinVar, of which 13 are classified as pathogenic and 1 as likely pathogenic. Five variants had not been reported before: p.K828fs in *ATM*, p.Y1527* in *ATR*, p.R89fs in *NBN*, p.Q244* in *PMS2* and p.A1653fs in *SLX4*.

**Table 2:** Rare protein truncating variants in the BROCA gene set

Gene	dbSNP ID	Variant type	Ref	Alt	Protein change	ClinVar	ExAC MAF (EUR)	CADD	Phenotype category
*ATM*		Frameshift deletion	A	–	p.K828fs	–	–	31	Non-aggressive
*ATM*	rs587779834	Frameshift deletion	G	–	p.V1268fs	5	4.5×10^−5^	28.3	Metastatic
*ATM*	rs786204751	Nonsense	C	T	p.Q1839*	4	–	46	Metastatic
*ATM*	rs770641163	Nonsense	C	T	p.R2993*	5|4	3.0×10^−5^	47	Metastatic
*ATR*		Nonsense	A	T	p.Y1527*	–	–	42	Metastatic
*BRCA2*	rs41293477	Nonsense	T	G	p.L1053*	5	–	29.2	Non-aggressive
*BRCA2*	rs80359454	Frameshift deletion	GAAA	–	p.E1493fs	5	–	29.6	Metastatic
*BRCA2*	rs80359470	Frameshift deletion	AA	–	p.N1626fs	5	–	25.5	Metastatic
*BRCA2*	rs11571658	Frameshift deletion	TT	–	p.L2092fs	5	1.5×10^−5^	24.6	Metastatic
*BRCA2*	rs80359752	Frameshift insertion	–	A	p.T3085fs	5	–	36	Metastatic
*CHEK2*	rs587781269	Nonsense	G	A	p.R95*	5	0	38	Non-aggressive
*NBN*	rs587780100	Frameshift deletion	TGTT	–	p.K233fs	5	4.7×10^−5^	35	Metastatic
*NBN*	rs587776650	Frameshift deletion	TTTGT	–	p.K219fs	5	3.2×10^−5^	35	Metastatic
*NBN*		Frameshift deletion	C	–	p.R89fs	–	–	8.3	Metastatic
*NBN*	rs587781891	Frameshift deletion	G	–	p.I41fs	5	–	28.3	Metastatic
*PALB2*	rs180177110	Nonsense	G	A	p.R753*	5	4.5×10^−5^	35	Metastatic
*PMS2*		Nonsense	G	A	p.Q244*	–	–	40	Metastatic
*PRSS1*	rs766199324	Frameshift deletion	C	–	p.P164fs	–	1.5×10^−5^	26	Metastatic
*RAD51D*	rs786202750	Frameshift deletion	A	–	p.T188fs	5	–	27	Metastatic
*SLX4*		Frameshift deletion	C	–	p.A1653fs	–	–	20.5	Non-aggressive
*SLX4*	rs767631456	Frameshift deletion	CT	–	p.S936fs	–	4.5×10^−5^	24	Metastatic
*XRCC2*	rs776336749	Frameshift deletion	T	–	p.H268fs	3	0	28.3	Metastatic

All Tier 1 variants identified for genes in the 60 gene BROCA panel are described, alongside case phenotype status. For each variant, the type of alteration, consequence at the protein level and CADD score are provided. dbSNP identifiers, ClinVar clinical significance score (5 = pathogenic, 4 = likely pathogenic, 3 = uncertain significance) and minor allele frequency in ExAC non-Finnish Europeans are provided for variants present in the respective databases

**Fig. 2:**
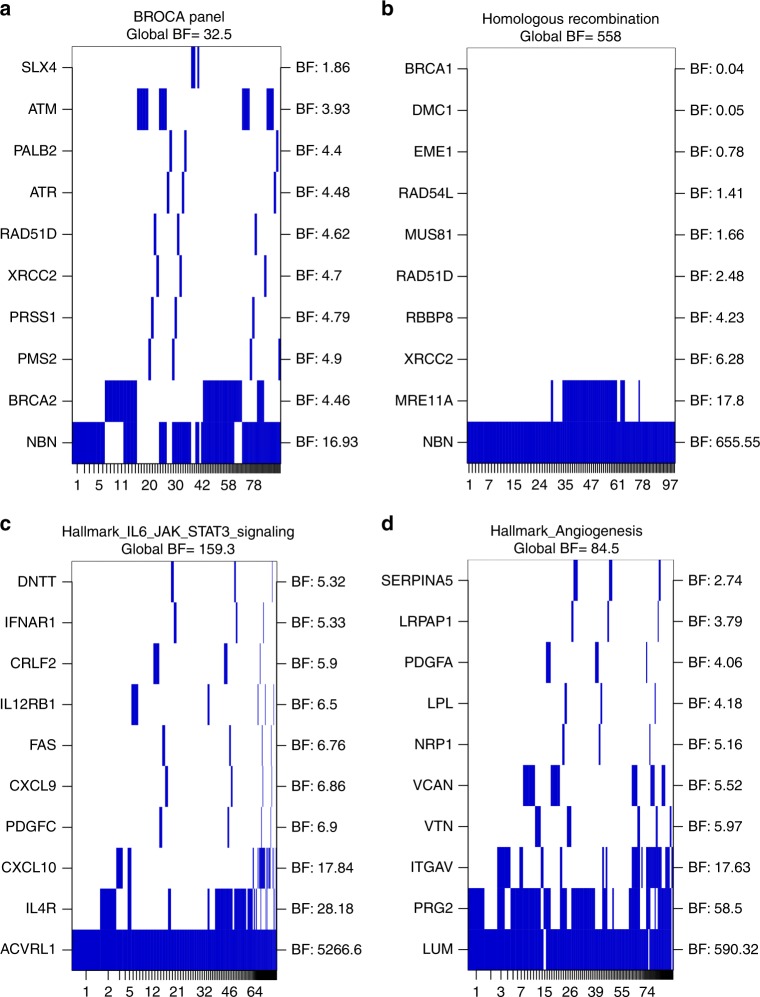
Bayesian Risk Index gene set analysis results. **a** BROCA gene set. **b** Homologous Recombination Pathway gene set. **c** Hallmark IL6/JAK/STAT Signalling gene set. **d** Hallmark Angiogenesis gene set. Analyses for differential burden of damaging variants between metastatic and non-aggressive cohorts were conducted for variants with MAF < 1% using Tier 1 variants only (BROCA gene set), or Tier 1 & 2 combined (Homologous Recombination Pathway and Hallmark gene sets). Each gene in the gene set was analysed as an individual region, with the top 10 genes included in the models depicted as rows in the plot, ordered according to gene level region Bayes Factor (BF). The gene regions or combinations of regions for the top models (indexed by the numbers on the *x*-axis) are plotted in the columns. These columns have widths proportional to and are ordered based on the posterior probability of the corresponding model. Global Bayes Factors for the entire gene set are indicated above each plot

We observed strong evidence for enrichment of Tier 1 variants within BROCA panel genes among cases with the aggressive phenotype using the Bayesian Risk Index method^30,31^ (Global BF = 32.5), which identified *NBN* and *BRCA2* as the genes contributing most strongly to this enrichment (Fig. 2a). Enrichment of PTVs among metastatic cases was confirmed by both SKAT-O (*P* *=* 0.004) and Fisher’s exact test (two-tailed, *P* *=* 0.003). This association remained with *BRCA2* excluded from the panel (Global BF = 25.9, SKAT-O *P* *=* 0.009). Carriers of BROCA gene PTVs also demonstrate reduced survival compared with non-carriers (*P* *=* 0.01, Supplementary Figure 3). When the BROCA panel analysis was expanded to include Tier 2 variants alongside Tier 1, evidence for differential burden between cohorts was no longer maintained (Global BF = 2.8).

24 of the 60 genes in the BROCA panel are DNA repair genes; of the 22 Tier 1 BROCA panel variants identified, 21 were within DNA repair genes and only 1 in the remainder of the panel. These DNA repair gene PTVs were identified across genes participating in a number of different DNA repair pathways, with the Fanconi Anaemia pathway, homologous recombination, DNA damage response and mismatch repair all represented. We therefore analysed an expanded set of 177 curated DNA repair genes,^33^ to assess whether rare variants in additional genes with a role in DNA repair may also contribute to predisposition towards an aggressive disease phenotype. We identified 64 rare Tier 1 variants in 47 different DNA repair genes in our cohort, with 23.7% of aggressive and 25.5% of non-aggressive cases harbouring one or more such variants. This expanded DNA repair gene set showed no overall evidence for association with the aggressive phenotype using either Bayesian Risk Index or SKAT-O methods, whether restricting the analyses to Tier 1 variants only (Global BF = 8.4, SKAT-O *P* = 0.66), or including both Tier 1 and Tier 2 (Global BF = 0.1, SKAT-O *P* = 0.19).

As no evidence for association with the metastatic phenotype was observed for the full set of curated DNA repair genes, we further stratified the analyses by the primary process or pathway each gene participates in. These analyses were performed for both Tier 1 and Tier 1 plus 2 variants separately. We observed very strong evidence for association between the homologous recombination pathway and the metastatic phenotype (Tier 1&2 Global BF = 558, Fig. 2b, Supplementary Table 3); this association was primarily driven by *NBN*, for which a Tier 1 or 2 variant was observed in 8 metastatic patients (5.8%) and no non-aggressive cases.

### Bayesian pathway analysis

To investigate additional biological processes that may contribute towards development of the aggressive phenotype, we performed a Bayesian Risk Index analysis using genes curated within the 50 MSigDB Hallmark gene set collections.^34^ We observed very strong evidence for differential burden of rare Tier 1 and Tier 2 variants in the HALLMARK_IL6_JAK_STAT3_SIGNALING gene set (genes up-regulated by IL6 via. STAT3; global BF = 159.3) and strong evidence for the HALLMARK_ANGIOGENESIS gene set (genes up-regulated during formation of blood vessels; global BF = 84.5). The genes contributing most strongly to these enrichments were *ACVRL1* and *LUM* respectively (Supplementary Table 4, Fig. 2c, d), with *ACVRL1* demonstrating a higher burden of damaging variants in non-aggressive cases and *LUM* enriched among the metastatic cohort (Supplementary Table 5). Within the HALLMARK_IL6_JAK_STAT3_SIGNALING gene set, genes with the highest burden of damaging variants in the metastatic cases were *IL1R2*, *IL9R*, *MAP3K8* and *TLR2*, with *ACVRL1* and *CD36* most enriched in non-aggressive samples. In the HALLMARK_ANGIOGENESIS gene set, *LUM*, *PRG2*, *ITGAV* and *VCAN* demonstrated the greatest enrichment in aggressive cases and *JAG1*, *CCND2* and *COL3A1* in non-aggressive cases (Supplementary Table 6). The majority of other Hallmark gene sets did not show clear evidence for association with aggressive status, although we observed positive association (BF = 3-20) for several additional processes (Supplementary Figure 4). Scrutiny of individual genes demonstrating strong evidence (Gene level BF ≥ 100) in the top models from any gene set analysis highlighted 8 further genes (*AXL*, *CHRNG*, *CP*, *DHRS2*, *MTOR*, *PARK2*, *RYR1*, *WISP2*) that may represent additional candidates for differential susceptibility towards metastatic PrCa (Supplementary Table 4, Supplementary Figure 4); the majority of which also have previously been linked to angiogenesis in the literature. These effects were primarily conferred through Tier 2 variants. Only two of the 10 genes identified through Bayesian Risk Index analysis were enriched for rare mutations in metastatic patients, with the remainder over represented for rare variants among cases with indolent disease (Supplementary Table 5).

### GO term enrichment analysis

To further interrogate wider biological processes that may contribute towards the development of the aggressive phenotype, we performed GO term enrichment analysis of genes with the largest OR calculated from the Tier 1 and Tier 2 variant burden. When including both genes with highest (>2.0) and lowest (<0.5) OR for metastatic disease, we observed a 1.9-fold enrichment of the term “extracellular matrix organisation” (GO:0030198, *P* *=* 0.025). Analysis of only the genes with lowest OR (higher burden of variants in the non-aggressive cases) showed a 9.0-fold enrichment of the term “fibril organisation” (GO:0097435, *P* *=* 0.035). Further investigation of the 8 genes contributing to this enrichment (*ADAMTS3*, *LTBP2*, *MFAP5*, *COL5A1*, *CD36*, *RIPK3*, *COL3A1* and *GSN*) revealed all to be involved in the collagen metabolism and angiogenesis pathways apart from *RIPK3*, which is involved in amyloid fibril formation (Supplementary Table 7). Similarly, restricting the analysis to only genes with high OR for aggressive PrCa revealed a 1.3-fold enrichment in the term “cellular component organisation” (GO:0016043, *P* *=* 0.012), a higher hierarchy term containing the “extracellular matrix organisation” category.

## Discussion

Prostate cancer demonstrates a heterogeneous clinical presentation; whilst the majority of patients present with intermediate phenotype and indolent disease, an appreciable subset progress to an advanced, poorer prognosis phenotype. Identification of more effective prognostic markers capable of distinguishing these outcomes would help inform clinical management pathways. Whole exome or alternatively whole genome sequencing provides great promise for the identification of novel genetic factors associated with PrCa aggressiveness, whether heritable or somatically acquired. Based on the relative rates of indolent and metastatic PrCa, higher penetrance germline variants predisposing towards greater likelihood of developing metastases in PrCa patients are likely to be predominantly rare. Extremely large sample sizes may therefore be required to achieve sufficient statistical power to reliably detect these associations; in most settings far beyond levels that are currently financially viable.^38^ Statistical power in modest sized rare variant sequencing studies of complex diseases may nevertheless be improved through the selection of enriched cohorts representing extreme phenotypes.^36^ Power can also be further improved through analysis of the effects of multiple aggregated rare variants within the same gene or sets of related genes.^38^ This approach does also have notable limitations however; particularly that the inclusion of a substantial proportion of benign variants or non-pathogenic genes in the analysis can reduce the signal to noise ratio, in turn potentially masking markers that are truly associated, and that curation of sets of interlinked genes relies on scholarly evidence that is inevitably incomplete and continually evolving. Restricting sequencing itself to smaller panels of candidate genes is another method frequently employed in sequencing studies; this simultaneously reduces both the cost of sequencing, thereby facilitating maximisation of sample size, and the magnitude of the multiple testing burden. In contrast to whole exome sequencing however, panel based approaches do not permit examination of all known genes, and therefore restrict investigations to plausible candidate genes based upon more narrow a priori hypotheses.

Our previous work has shown that the BROCA cancer risk gene panel, designed primarily to evaluate hereditary breast and ovarian cancer predisposition, is informative in stratifying PrCa risk among individuals with strong family history of PrCa.^11^ Through the case–case analysis presented in this study, we further demonstrate that rare germline PTVs in the now extended BROCA panel are also enriched in metastatic patients compared with PrCa cases with a non-aggressive clinical presentation. Furthermore, these mutations were observed almost exclusively in the DNA repair gene component of the panel. Overall, 12.2% of the metastatic cases examined carried a protein truncating mutation in a BROCA panel gene compared with 2.8% of non-aggressive cases, with *BRCA2*, *ATM* and *NBN* (frequencies 2.9%, 2.2% and 2.2% in metastatic patients respectively) being the most commonly mutated genes among the aggressive cohort. The findings for *BRCA2* and *ATM* are largely in agreement with recent candidate gene studies in castration resistant metastatic PrCa cases^12,39^ and lethal PrCa cohorts^13^ of primarily European ancestry. The Bayesian Risk Index method that we employed ranked *NBN* as the gene within the BROCA panel for which PTVs contributed most strongly towards the metastatic phenotype. Furthermore, separate analyses of individual DNA repair pathways indicated a likely additional contribution by rare non-truncating *NBN* variants to increased risk of metastases. These findings are consistent with previous observations that carriers of a Slavic founder mutation (rs587776650 / 657del5) in *NBN*^40^ experience significantly higher PrCa mortality,^41^ and demonstrates that additional rare *NBN* variants, including missense variants, may increase the risk of aggressive PrCa in the British population.

Although this study therefore provides further evidence that germline variants within a handful of specific DNA repair genes increase risk of aggressive PrCa,^9–13,39^ no significant association of rare truncating variants with metastatic disease was observed in our analysis of an expanded set of 177 genes curated specifically as contributing towards DNA repair.^33^ This suggests that only a subset of specific DNA repair genes or pathways contribute substantially to the predisposition towards the poor prognosis phenotype, and that germline PTVs within any family members for which association has not yet been established are likely to occur at extremely low frequencies in the British population. The PTVs we identified were observed across genes contributing to multiple separate DNA repair pathways, in particular homologous recombination and the Fanconi anaemia pathway. Stratification of the DNA repair gene set by pathway provided strong evidence for a role by homologous recombination in increased susceptibility to development of metastases, with the association driven primarily by *NBN*.

Whilst we observed a high rate of germline DNA repair gene mutations among our metastatic cohort in comparison to non-aggressive PrCa cases, the absence of these mutations in the majority of the cohort implies that additional biological processes, besides faulty DNA damage response and repair, could also be instrumental in predisposition towards or protection against metastatic disease. Rare germline coding variants in *TET2* have previously been reported to be enriched in aggressive PrCa cases of African ancestry; however this association was ethnicity specific and was not found for European ancestry individuals.^42^ Within our cohort, we observed truncating *TET2* mutations in 3 patients, all non-aggressive, whilst Tier 2 variants were identified in one additional metastatic and one non-aggressive case. A low frequency missense variant in *HOXB13* (rs138213197 / G84E) has also been robustly demonstrated to increase risk of PrCa in European ancestry populations and is associated with familial clustering and younger age of diagnosis; however no association with clinical presentation or survival has been demonstrated.^43–45^ The *HOXB13* G84E variant was identified in 1 non-aggressive case only in our study and no further Tier 1 or 2 *HOXB13* variants were identified within either cohort. These observations provide further support that these two genes are unlikely to contribute substantially towards increased risk of aggressive disease in European ancestry populations.

We conducted further gene set analyses in an attempt to identify additional genes that may influence prognosis in PrCa patients. GO term enrichment analysis on genes displaying a differential case–case burden of rare disruptive variants was performed, which highlighted genes involved in extracellular matrix organisation and remodelling, a key event in angiogenesis.^46^ Interestingly, we also found damaging variants in genes associated with collagen fibril formation enriched among the non-aggressive cases, alluding to a possible protective function against tumour invasiveness of certain defects in the extracellular matrix organisation. In line with these observations, Bayesian Risk Index analysis of hallmark gene sets in MSigDB demonstrated evidence that disruptive germline variants in genes contributing towards angiogenesis can distinguish patients with metastatic and localised disease. This analysis selected two gene sets with high evidence of differential burden between the extreme phenotypes and highlighted two genes in particular driving these associations, *LUM* and *ACVRL1*. Further inclusion of genes that demonstrated a gene-level BF > 100 within any gene set analysis returns a total of 10 genes for consideration. The majority of these genes have previously been reported to be linked to angiogenesis, proliferation and poor prognosis in multiple cancer types, especially through modulation of TGF-ß signalling. Indeed, pharmacological inhibitors of two genes (*ACVRL1* and *AXL*) are currently the subject of clinical trials in a range of tumour types as prospective anti-angiogenic therapeutics.^47,48^

In keeping with the role of many of these genes in developmental or homoeostatic processes, PTVs were observed in only 2 of the 10 genes shortlisted through the Bayesian Risk Index analysis, with Tier 2 variants accounting for 81 of the 88 rare variants identified in these genes in our cohort. We also observed differential enrichment of mutations within either the metastatic or non-aggressive cohort for different genes, implying a combination of potential deleterious and protective effects towards tumour invasiveness. Two genes exhibited enrichment of disruptive mutations in metastatic cases, *LUM* and *AXL*. *LUM* codes for lumican, a collagen-binding protein found in interstitial collagenous matrices throughout the body and implicated as an inhibitor of angiogenesis^49,50^; disruptive mutations within *LUM* would therefore be expected to facilitate tumour vasculature formation. Suppression of *AXL* expression has been reported to play a central role in the proliferation of disseminated PrCa cells in bone marrow into metastatic lesions from a dormant state,^51^ representing a plausible route through which disruptive variants in *AXL* could predispose individuals that develop PrCa towards a metastatic phenotype. The remainder of genes identified were enriched for mutations in non-aggressive rather than metastatic cases, with many also demonstrating biologically plausible rationale for how these variants could potentially confer a protective effect against tumour migration. *ACVRL1* (also known as *ALK1*) is a cell surface receptor in the TGF-beta signalling pathway which binds bone morphogenic protein (BMP)-9 and -10, appears to be involved in developmental and angiogenic blood vessel formation, and has been shown to be highly expressed in the vasculature and stroma in a high proportion of prostate tumours.^52^ Germline disruptive variants within *ACVRL1* could therefore in principle inhibit development of new tumour vasculature and hinder the onset of metastases. Similarly, mTOR is a known promoter of angiogenesis through the VEGF signalling pathway and is frequently targeted for inhibition in human tumours to exert anti-angiogenic activity.^53^ Also of note is the *CP* gene, which codes for ceruloplasmin, the major copper-carrying protein in the blood. Association between angiogenesis and copper levels has been established, with elevated levels of ceruloplasmin itself linked to development of metastases in rabbit models.^54^ Disruptive mutations in *CP* could therefore feasibly be protective against angiogenesis in PrCa. Other genes enriched for mutations in patients with indolent disease include *WISP2*, a gene that inhibits proliferation of vascular smooth muscle cells and has been associated with invasion, metastasis and poor prognosis, although opposing effects have been reported for different cancer types,^55,56^ *DHRS2*, which stabilises p53 by inhibiting Mdm2 and may be upregulated in breast tumour endothelium in comparison to normal breast vasculature,^57,58^ and *PARK2*, a gene linked to hereditary Parkinson’s disease that also demonstrates tumour suppressor functions and might confer an influence on apoptosis through modulation of mitophagy.^59,60^

The use of differential ages in the definition of our aggressive and non-aggressive cohorts enabled us to maximise our power to detect associations through the analysis of sample groups at the extremes of the phenotypic spectrum of PrCa, however could also represent a potential confounder. As 105 of the 120 deaths within our sample cohort were PrCa specific, and no cause was recurrent among the remaining 15 deaths, there would appear to be minimal evidence of any overlapping associations with large effect sizes against other disease phenotypes within our data. We cannot completely exclude however that this could represent a potential limitation of our study, whilst the use of age as a criterion in the definition of aggressiveness also limits our ability to examine the effect of mutation carrier status on age at onset; since carrier status, aggressiveness, and age are all correlated. Nonetheless, this study demonstrates that germline PTVs in specific DNA repair genes occur significantly more frequently among, and therefore can be used to distinguish, PrCa patients likely to progress to metastatic disease. In particular, mutations in the *NBN, BRCA2* and *ATM* genes are associated with development of an aggressive clinical phenotype. Using gene-set enrichment analyses, we have also found strong preliminary evidence that rare, predominantly non-truncating germline variants predicted to affect protein function in specific genes contributing to angiogenesis are implicated in conferring either predisposition towards, or protection against, progression to metastatic disease in men who develop PrCa. If validated in larger independent cohorts, or meta-analyses of multiple studies, these genes would represent prognostic markers that may aid identification of PrCa patients at high or low risk of developing invasive disease, who would warrant distinct clinical management pathways. These genes could also represent exciting novel targets for targeted therapies tailored towards individual PrCa patients with aggressive phenotype. These initial findings therefore warrant follow-up in larger cohorts to validate their potential role in predisposition toward metastatic disease.

## Electronic supplementary material

Supplementary Figure 1

Supplementary Figure 2

Supplementary Figure 3

Supplementary Figure 4

Supplementary Table 1

Supplementary Table 2

Supplementary Table 3

Supplementary Table 4

Supplementary Table 5

Supplementary Table 6

Supplementary Table 7

## Availability of data and material

The data generated during the current study are available from the corresponding author on reasonable request.
