# Correction: Rare germline variants in DNA repair genes and the angiogenesis pathway predispose prostate cancer patients to develop metastatic disease

**DOI:** 10.1038/s41416-019-0419-4

**Published:** 2019-03-06

**Authors:** Martina Mijuskovic, Edward J. Saunders, Daniel A. Leongamornlert, Sarah Wakerell, Ian Whitmore, Tokhir Dadaev, Clara Cieza-Borrella, Koveela Govindasami, Mark N. Brook, Christopher A. Haiman, David V. Conti, Rosalind A. Eeles, Zsofia Kote-Jarai

**Affiliations:** 1 Oncogenetics, Division of Genetics and Epidemiology, The Institute of Cancer Research, London, SW7 3RP UK; 2 Department of Preventive Medicine, Keck School of Medicine, University of Southern California/Norris Comprehensive Cancer Center, Los Angeles, CA 90015 USA; 3 The Royal Marsden NHS Foundation Trust, London, SW3 6JJ UK

**Keywords:** Cancer genetics, Cancer genetics

**Correction to:** *British Journal of Cancer* (2018) **119**:96–104; 10.1038/s41416-018-0141-7; http://www.bjcancer.com; published online 19 June 2018

This article was originally published under the standard License to Publish, but has now been made available under a CC BY 4.0 license. The PDF and HTML versions of the paper have been modified accordingly.

