# Supplementary material for: Rare germline variants in DNA repair genes and the angiogenesis pathway predispose prostate cancer patients to develop metastatic disease
Source: Br J Cancer. 2018 Jun 19;119(1):96–104. doi: 10.1038/s41416-018-0141-7 (PMC6035259; doi:10.1038/s41416-018-0141-7)
Supplement: Supplementary file 1 — Supplementary Figure 1 [file 41416_2018_141_MOESM1_ESM.pdf]

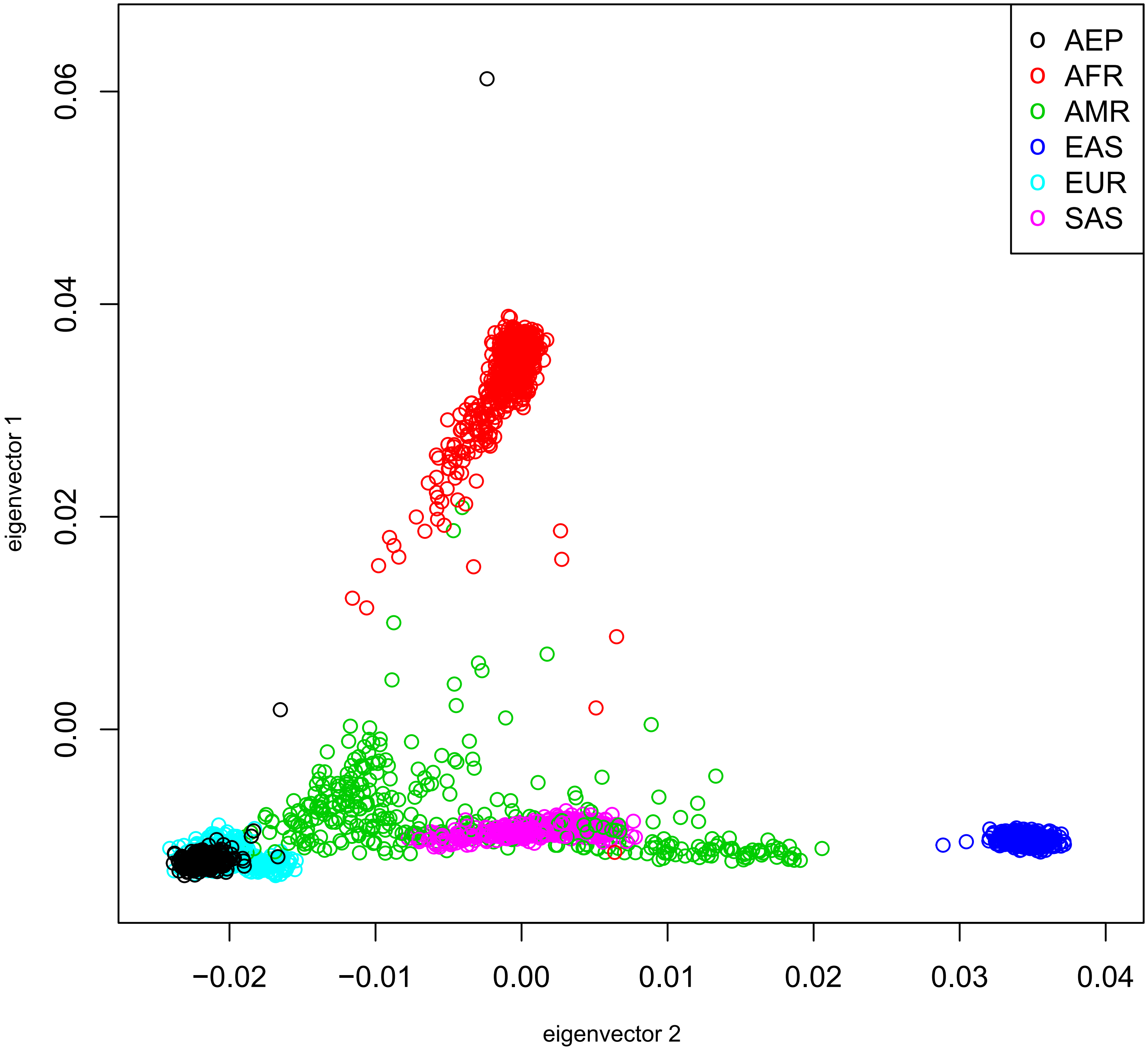

Supplementary Figure 1 – Ancestry of samples relative to 1000 Genomes Phase 3 populations. Principal Component Analysis depicting genetic ancestry of our full study cohort of self-reported European ancestry individuals unstratified for aggressive status (AEP) compared to individuals from the five 1000 Genomes super populations; African (AFR), Ad-mixed American (AMR), East Asian (EAS), European (EUR), South Asian (SAS).
