# Supplementary material for: Rare germline variants in DNA repair genes and the angiogenesis pathway predispose prostate cancer patients to develop metastatic disease
Source: Br J Cancer. 2018 Jun 19;119(1):96–104. doi: 10.1038/s41416-018-0141-7 (PMC6035259; doi:10.1038/s41416-018-0141-7)
Supplement: Supplementary file 2 — Supplementary Figure 2 [file 41416_2018_141_MOESM2_ESM.pdf]

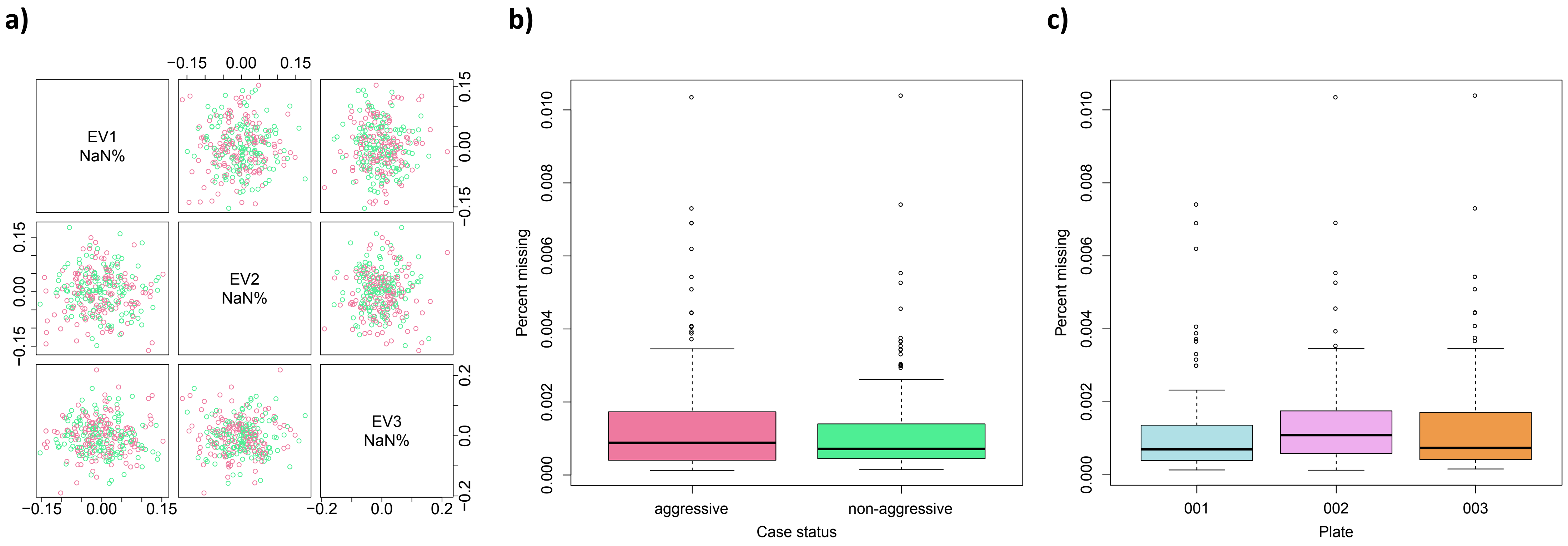

Supplementary Figure 2 – Evaluation of samples for potential batch effect confounders. a) Principal Component Analysis highlighted by case status. Pairs for the first three Principal Components are shown for non-aggressive (green) and aggressive (pink) samples. **b)** Assessment of genotype call rates by case status. **c)** Assessment of genotype call rate by sequencing library preparation workflow plate. No evidence for underlying genetic or artefactual confounding effects was observed between or within phenotype groups.
