# Supplementary material for: Rare germline variants in DNA repair genes and the angiogenesis pathway predispose prostate cancer patients to develop metastatic disease
Source: Br J Cancer. 2018 Jun 19;119(1):96–104. doi: 10.1038/s41416-018-0141-7 (PMC6035259; doi:10.1038/s41416-018-0141-7)
Supplement: Supplementary file 3 — Supplementary Figure 3 [file 41416_2018_141_MOESM3_ESM.pdf]

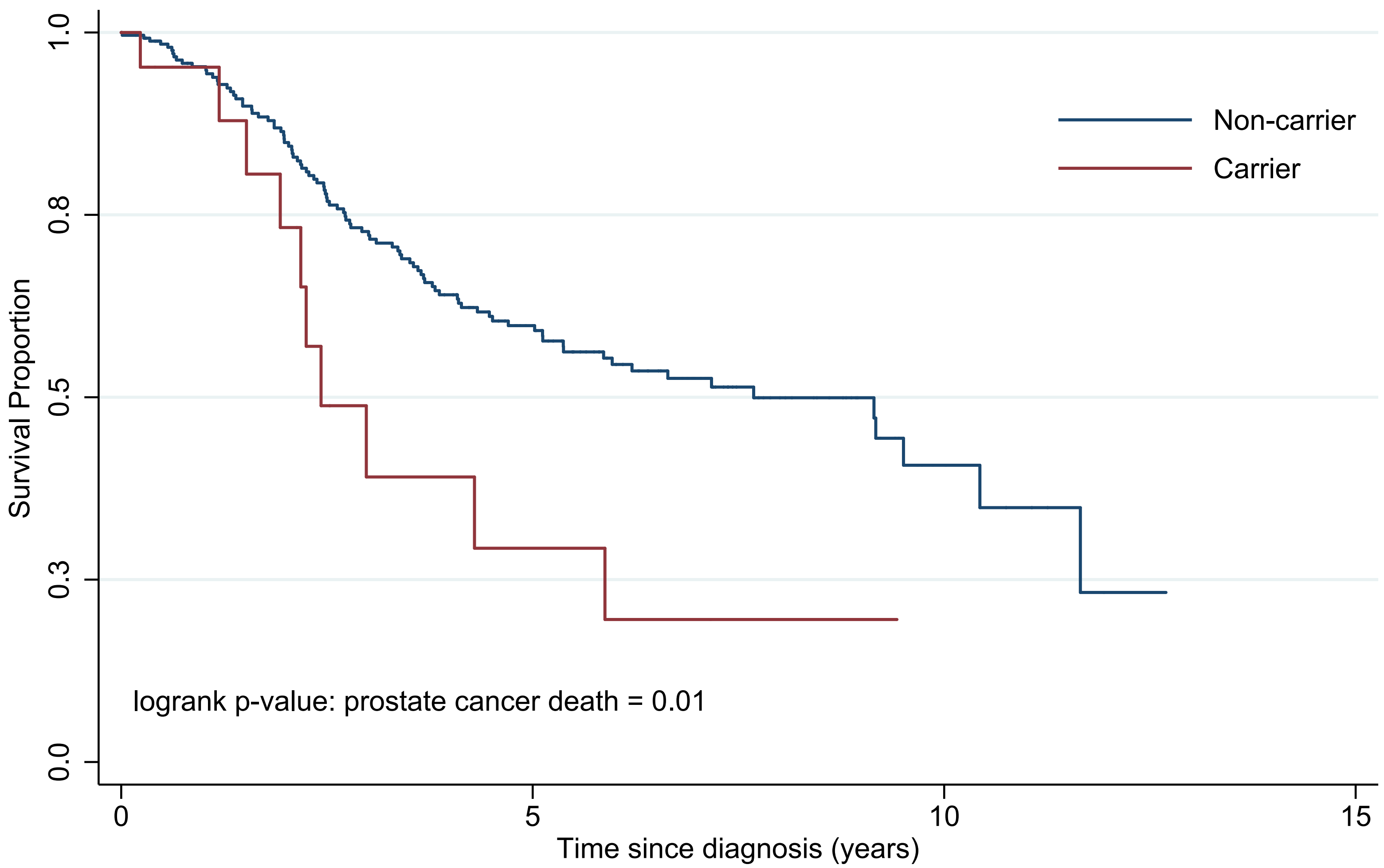

Supplementary Figure 3 - Survival analysis for BROCA gene set mutation carriers and non-carriers. Kaplan-Meier curve depicting the relative PrCa specific survival of BROCA panel Tier 1 mutation carriers (red line) and non-carriers (blue line). Survival analyses were performed agnostic to aggressive status.
