# Supplementary material for: Rare germline variants in DNA repair genes and the angiogenesis pathway predispose prostate cancer patients to develop metastatic disease
Source: Br J Cancer. 2018 Jun 19;119(1):96–104. doi: 10.1038/s41416-018-0141-7 (PMC6035259; doi:10.1038/s41416-018-0141-7)
Supplement: Supplementary file 4 — Supplementary Figure 4 [file 41416_2018_141_MOESM4_ESM.pdf]

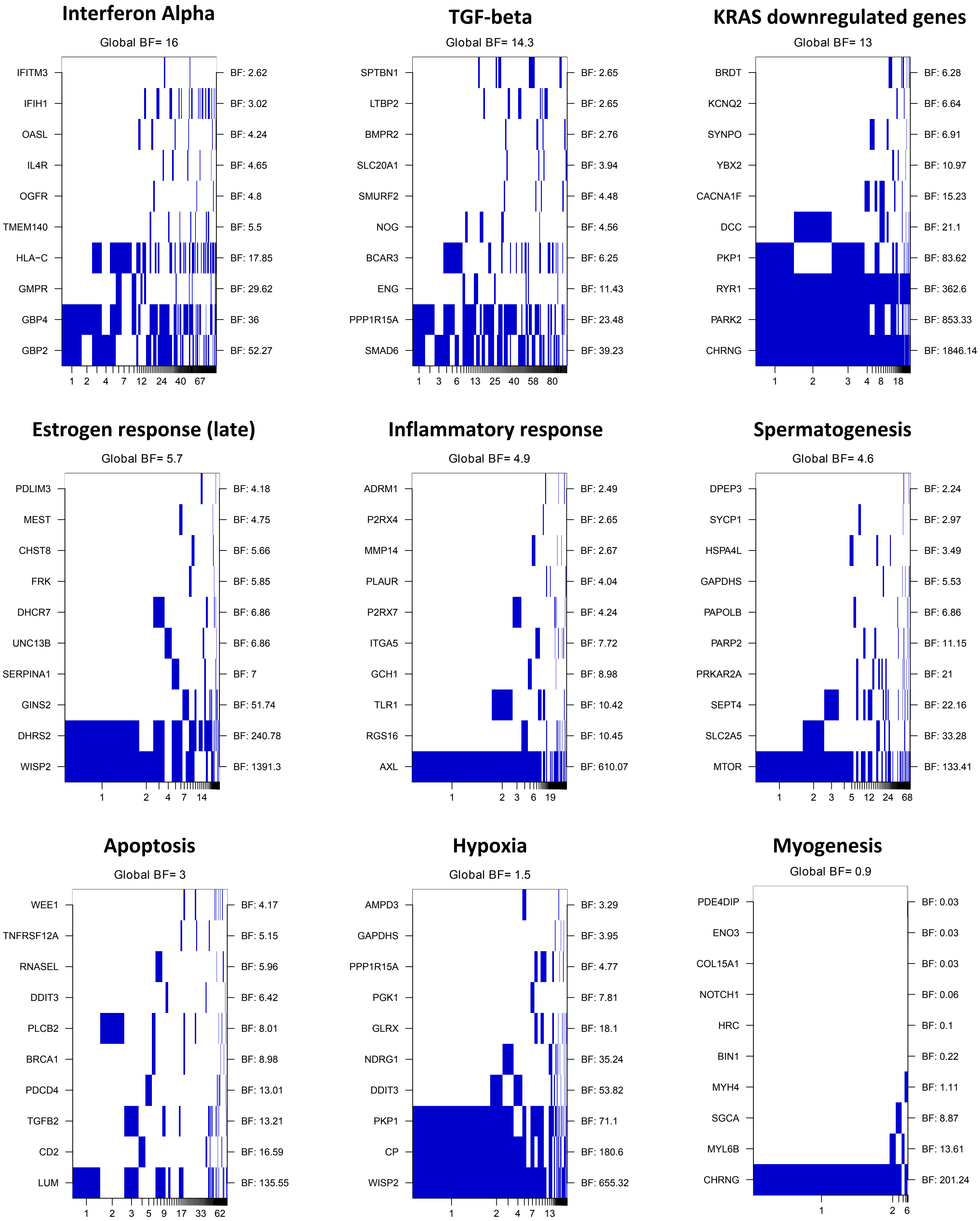

Supplementary Figure 4 - Results of Bayesian Risk Index analysis of Hallmark Gene Sets. Only plots for gene sets with a global Bayes Factor (BF) >10 or in which any individual gene achieved BF ≥100 are shown (IL6_JAK_STAT3_SIGNALING and ANGIOGENESIS gene sets are instead shown in Figure 2). Analyses for differential burden of damaging variants between metastatic and non-aggressive cohorts were conducted for Tier 1 & 2 variants combined with MAF <1%. Each gene in the gene set was analysed as an individual region, with the top 10 genes included in the models depicted as rows in the plot, ordered according to gene level region BF. The gene regions or combinations of regions for the top models (indexed by the numbers on the x-axis) are plotted in the columns. These columns have widths proportional to and are ordered based on the posterior probability of the corresponding model. Global Bayes Factors for the entire gene set are indicated above each plot.
