# Supplementary material for: Rare germline variants in DNA repair genes and the angiogenesis pathway predispose prostate cancer patients to develop metastatic disease
Source: Br J Cancer. 2018 Jun 19;119(1):96–104. doi: 10.1038/s41416-018-0141-7 (PMC6035259; doi:10.1038/s41416-018-0141-7)
Supplement: Supplementary file 5 — Supplementary Table 1 [file 41416_2018_141_MOESM5_ESM.pdf]

a)

Exclusion criteria	Variants excluded	Variants remain
Non-exonic or splicing	506,198	174,815
>5% missing data	5312	169,503
GATK VQSR (99.9 level)	2183	167,320
Monomorphic	1445	165,875
<95% samples with Genotype Quality ≥20	3772	162,103
<95% samples with Depth ≥8	3780	158,323
**Total**	**522,690**	**158,323**

b)

Exclusion criteria	Samples excluded	Samples remain
Low coverage (<80% at 20×)	2	286
Contamination (>10%)	2	284
>3 × SD heterozygosity	2	282
Mixed ancestry (and >3 × SD heterozygosity)	2	280
**Total**	**8**	**280**

c)

Exclusion criteria	Variants excluded	Variants remain
All genotypes as reference	6586	151,737
Monomorphic	175	151,562
>5% missing data	236	151,326
HWE ($p < 10^{-5}$)	539	150,787
**Total**	**7536**	**150,787**

Supplementary Table 1 – Sample and variant QC procedures. a) Variant filtering pre-sample QC. QC is first performed at the variant level to remove low quality variants prior to sample level QC procedures. All variants situated outside of coding regions or splice sites, with missing genotype data in >5% samples, predicted as false positives by GATK's VQSR (variant quality score recalibration), that were monomorphic, that had low quality scores in >5% samples or had low depth of sequencing coverage in >5% samples were excluded. **b) Sample QC.** Sample QC was performed using the filtered variant catalogue generated through pre-sample QC. Samples with low coverage of target regions, appreciable contamination, high heterozygosity or mixed ancestry were excluded from further analysis. **c) Variant filtering post-sample QC.** Additional variant filtering was performed using the final set of quality filtered samples to exclude variants that were reference-only, monomorphic, had >5% missing genotype data or deviated from Hardy-Weinberg equilibrium in the final sample cohort.
