# Supplementary material for: Rare germline variants in DNA repair genes and the angiogenesis pathway predispose prostate cancer patients to develop metastatic disease
Source: Br J Cancer. 2018 Jun 19;119(1):96–104. doi: 10.1038/s41416-018-0141-7 (PMC6035259; doi:10.1038/s41416-018-0141-7)
Supplement: Supplementary file 6 — Supplementary Table 2 [file 41416_2018_141_MOESM6_ESM.pdf]

a)

Classification	Description	Total Variants	MAF <1%
Tier 1	PTVs (Frameshift, nonsense, STOP insertion and splice variants)	4924	4240
Tier 2	Non-PTVs with CADD score ≥20	39,080	31,372
Tier 3	All other variants	106,783	62,188
		**150,787**	**97,800**

b)

Variant Frequency Category	Total Variants
Common (MAF ≥5%)	33,663
Low frequency (MAF 1-5%)	19,324
Rare (MAF <1%)	82,333
Not observed in ExAC	15,467
**Total**	**150,787**

Supplementary Table 2 – Predicted functional impact of variants passing QC. a) Variant classification by predicted functional consequence. Variants were classified into 3 categories according to their predicted functional effects. Tier 1 contains variants predicted as deleterious and comprises protein truncating variants (PTVs). Tier 2 contains variants that are predicted as damaging and comprises non-truncating variants with CADD score ≥20. Tier 3 contains all other variants and variants in this category were excluded during downstream analyses. Rare variants were designated as those with a minor allele frequency (MAF) <1% in non-Finnish Europeans in the ExAC database. **b) Frequency distribution of variants in non-Finnish Europeans in the ExAC database.** The majority of coding variants identified in our sample cohort were low frequency, with approximately 10% not observed in the ExAC database.
