# Supplementary material for: Rare germline variants in DNA repair genes and the angiogenesis pathway predispose prostate cancer patients to develop metastatic disease
Source: Br J Cancer. 2018 Jun 19;119(1):96–104. doi: 10.1038/s41416-018-0141-7 (PMC6035259; doi:10.1038/s41416-018-0141-7)
Supplement: Supplementary file 7 — Supplementary Table 3 [file 41416_2018_141_MOESM7_ESM.pdf]

DNA Repair Pathway	Tier 1 BF	Tier 1&2 BF	Genes BF >100 in either analysis
Base Excision Repair (BER)	2.4	7.6	
DNA damage disease linked	1.7	7.4	
DNA damage response	3.8	7.8	
DNA polymerases	0[$]	8.3	
Fanconi Anemia (FA)	6.2	19.5	
Homologous Recombination (HR)	7.3	558	*NBN* (BF = 655.55)
Mismatch Repair (MMR)	0[$]	18	
Nucleotide Excision Repair (NER)	8.6	10.9	
Non-Homologous End Joining (NHEJ)	0[$]	1.5	
Poly ADP-Ribose Polymerase (PARP)	n/a[&]	3.7	

[$] All models in the model space enumerated due to low numbers of Tier 1 variants in the pathway
[&] No Tier 1 variants were present in any genes within this pathway

Supplementary Table 3 – Results of Bayesian Risk Index analysis of Individual DNA Repair Pathways. The global Bayes Factor (BF) for each gene set are listed for Tier 1 only or Tier 1 & 2 variants combined, alongside individual genes with BF ≥100 in either variant set analysis.
