# Supplementary material for: Rare germline variants in DNA repair genes and the angiogenesis pathway predispose prostate cancer patients to develop metastatic disease
Source: Br J Cancer. 2018 Jun 19;119(1):96–104. doi: 10.1038/s41416-018-0141-7 (PMC6035259; doi:10.1038/s41416-018-0141-7)
Supplement: Supplementary file 8 — Supplementary Table 4 [file 41416_2018_141_MOESM8_ESM.pdf]

Hallmark Gene Set	Global BF	Genes with Gene Level BF ≥100
IL6_JAK_STAT3_SIGNALING	159.3	*ACVRL1* (BF = 5266.6)
ANGIOGENESIS	84.5	*LUM* (BF = 590.3)
INTERFERON_ALPHA_RESPONSE	16.0	
TGF_BETA_SIGNALING	14.3	
KRAS_SIGNALING_DN	13.0	*CHRNG* (BF = 1846.1); *PARK2* (BF = 853.3); *RYR1* (BF = 362.6)
PANCREAS_BETA_CELLS	8.6	
NOTCH_SIGNALING	8.5	
REACTIVE_OXIGEN_SPECIES_PATHWAY	6.8	
CHOLESTEROL_HOMEOSTASIS	6.1	
ESTROGEN_RESPONSE_LATE	5.7	*WISP2* (BF = 1391.3); *DHRS2* (BF = 240.8)
WNT_BETA_CATENIN_SIGNALING	5.5	
MYC_TARGETS_V2	5.3	
HEDGEHOG_SIGNALING	5.2	
INFLAMMATORY_RESPONSE	4.9	*AXL* (BF = 610.1)
SPERMATOGENESIS	4.6	*MTOR* (BF = 133.4)
APICAL_SURFACE	4.4	
PEROXISOME	3.4	
ANDROGEN_RESPONSE	3.4	
PI3K_AKT_MTOR_SIGNALING	3.2	
APOPTOSIS	3.0	*LUM* (BF = 135.6)
UNFOLDED_PROTEIN_RESPONSE	2.9	
PROTEIN_SECRETION	2.5	
COAGULATION	2.1	
DNA_REPAIR	1.8	
ALLOGRAFT_REJECTION	1.7	
UV_RESPONSE_UP	1.6	
HYPOXIA	1.5	*WISP2* (BF = 655.3); *CP* (BF = 180.6)
OXIDATIVE_PHOSPHORYLATION	1.3	
TNFA_SIGNALING_VIA_NFKB	1.3	
MYC_TARGETS_V1	1.2	
BILE_ACID_METABOLISM	1.2	
P53_PATHWAY	1.1	
MYOGENESIS	0.9	*CHRNG* (BF = 201.2)
E2F_TARGETS	0.7	
INTERFERON_GAMMA_RESPONSE	0.7	
FATTY_ACID_METABOLISM	0.7	
MTORC1_SIGNALING	0.7	
COMPLEMENT	0.6	
UV_RESPONSE_DN	0.5	
IL2_STAT5_SIGNALING	0.3	
KRAS_SIGNALING_UP	0.3	
G2M_CHECKPOINT	0.3	
HEME_METABOLISM	0.3	
ADIPOGENESIS	0.2	
GLYCOLYSIS	0.1	
ESTROGEN_RESPONSE_EARLY	0.1	
XENOBIOTIC_METABOLISM	0.1	
APICAL_JUNCTION	0.0	
MITOTIC_SPINDLE	0.0	
EPITHELIAL_MESENCHYMAL_TRANSITION	0.0	

Supplementary Table 4 – Results of Bayesian Risk Index analysis of Hallmark Gene Sets. The global Bayes Factor (BF) for each gene set and individual genes with BF ≥100 in any analysis are listed.
