# Supplementary material for: Rare germline variants in DNA repair genes and the angiogenesis pathway predispose prostate cancer patients to develop metastatic disease
Source: Br J Cancer. 2018 Jun 19;119(1):96–104. doi: 10.1038/s41416-018-0141-7 (PMC6035259; doi:10.1038/s41416-018-0141-7)
Supplement: Supplementary file 9 — Supplementary Table 5 [file 41416_2018_141_MOESM9_ESM.pdf]

	Tier 1 & 2		Tier 1 only	
**Gene**	**Aggressive**	**Non- aggressive**	**Aggressive**	**Non-aggressive**
ACVRL1	0	3	0	0
AXL	10	1	0	0
CHRNG	3	6	1	0
CP	0	5	0	0
DHRS2	3	12	0	0
LUM	5	0	0	0
MTOR	1	8	0	0
PARK2	0	6	0	0
RYR1	10	14	3	3
WISP2	0	1	0	0

Supplementary Table 5 – Burden of mutations between metastatic and non-aggressive cohorts for genes with BF ≥100 in the Bayesian Risk Index analysis of Hallmark Gene Sets. Counts of Tier 1 variants, or Tier 1 and 2 variants combined, stratified by aggressiveness status. The majority of rare disruptive variants within the genes identified through this analysis were non-protein truncating, and were enriched in the non-aggressive cohort, implying a protective effect against the likelihood of developing metastatic PrCa.
