# Supplementary material for: Rare germline variants in DNA repair genes and the angiogenesis pathway predispose prostate cancer patients to develop metastatic disease
Source: Br J Cancer. 2018 Jun 19;119(1):96–104. doi: 10.1038/s41416-018-0141-7 (PMC6035259; doi:10.1038/s41416-018-0141-7)
Supplement: Supplementary file 10 — Supplementary Table 6 [file 41416_2018_141_MOESM10_ESM.pdf]

HALLMARK_IL6_JAK_STAT3_SIGNALING

	Mutation Count		
Gene	Non-aggressive	Aggressive	Odds Ratio
ACVRL1	3	0	0.14
CD36	5	1	0.20
CRLF2	2	0	0.20
CXCL10	2	0	0.20
IL12RB1	2	0	0.20
A2M	4	1	0.25
PIK3R5	4	1	0.25
IL4R	3	1	0.33
CXCL9	1	0	0.34
DNTT	1	0	0.34
FAS	1	0	0.34
IFNAR1	1	0	0.34
IRF9	1	0	0.34
PDGFC	1	0	0.34
HAX1	1	2	2.04
HMOX1	1	2	2.04
IFNGR2	1	2	2.04
IL6ST	1	2	2.04
CBL	0	1	3.06
CCL7	0	1	3.06
CD38	0	1	3.06
CD44	0	1	3.06
CSF1	0	1	3.06
CSF2RB	0	1	3.06
IFNGR1	0	1	3.06
IL18R1	0	1	3.06
STAM2	0	1	3.06
TGFB1	0	1	3.06
TNF	0	1	3.06
TNFRSF12A	0	1	3.06
INHBE	1	3	3.09
IL1R2	0	2	5.15
IL9R	0	2	5.15
MAP3K8	0	2	5.15
TLR2	0	2	5.15

HALLMARK_ANGIOGENESIS

	Mutation Count		
Gene	Non-aggressive	Aggressive	Odds Ratio
JAG1	3	0	0.14
CCND2	2	0	0.20
COL3A1	4	1	0.25
PTK2	1	0	0.34
APOH	2	1	0.50
THBD	2	1	0.50
CXCL6	1	2	2.04
JAG2	1	2	2.04
SERPINA5	1	2	2.04
LPL	0	1	3.06
LRPAP1	0	1	3.06
NRP1	0	1	3.06
PDGFA	0	2	5.15
VTN	0	2	5.15
VCAN	1	6	6.32
ITGAV	0	3	7.26
PRG2	0	4	9.40
LUM	0	5	11.57

Supplementary Table 6 – Burden of predicted disruptive mutations in genes curated in the IL6/JAK/STAT signalling and angiogenesis Hallmark gene sets. The number of rare (MAF ≤1%) Tier 1 and 2 variants identified within non-aggressive and metastatic cases are listed for genes with odds ratio ≥2 or ≤0.5. For genes with no mutations identified in one cohort, odds ratios were estimated through addition of 0.5 to the mutation count for both phenotype categories.
