# Supplementary material for: Rare germline variants in DNA repair genes and the angiogenesis pathway predispose prostate cancer patients to develop metastatic disease
Source: Br J Cancer. 2018 Jun 19;119(1):96–104. doi: 10.1038/s41416-018-0141-7 (PMC6035259; doi:10.1038/s41416-018-0141-7)
Supplement: Supplementary file 11 — Supplementary Table 7 [file 41416_2018_141_MOESM11_ESM.pdf]

Gene	GO term annotations	Function
ADAMTS3	collagen fibril organization, vascular endothelial growth factor production	A disintegrin and metalloproteinase with thrombospondin motifs 3
LTBP2	extracellular fibril organization	Latent-transforming growth factor beta-binding protein 2
MFAP5	extracellular matrix organization, extracellular fibril organization	Microfibrillar-associated protein 5
COL5A1	blood vessel development, cell adhesion, cell migration, extracellular matrix organization, collagen fibril organization	Collagen alpha-1(V) chain
CD36	positive regulation of cell-matrix adhesion	Platelet glycoprotein 4
RIPK3	amyloid fibril formation, necroptotic process	Receptor-interacting serine/threonine-protein kinase 3
COL3A1	cell-matrix adhesion, extracellular matrix organization, collagen fibril organization	Collagen alpha-1(III) chain
GSN	actin filament polymerization, regulation of wound healing, spreading of epidermal cells	Gelsolin

Supplementary Table 7 – Description of genes annotated with GO term "fibril organization".
